# The Implementation of a Text Messaging Intervention to Improve HIV Continuum of Care Outcomes Among Persons Recently Released From Correctional Facilities: Randomized Controlled Trial

**DOI:** 10.2196/16220

**Published:** 2020-02-13

**Authors:** Breana J Uhrig Castonguay, Andrew E Cressman, Irene Kuo, Rudy Patrick, Claudia Trezza, Alice Cates, Halli Olsen, James Peterson, Ann Kurth, Lauri B Bazerman, Curt G Beckwith

**Affiliations:** 1 University of North Carolina Center for AIDS Research Lineberger Cancer Center University of North Carolina at Chapel Hill Chapel Hill, NC United States; 2 The Center for Prisoner Health and Human Rights Providence, RI United States; 3 Brown University School of Public Health Providence, RI United States; 4 Milken Institute School of Public Health George Washington University Washington, DC United States; 5 District of Columbia Center for AIDS Research Washington, DC United States; 6 Division of Infectious Diseases and Global Public Health Department of Medicine University of California, San Diego La Jolla, CA United States; 7 Yale University School of Nursing New Haven, CT United States; 8 The Miriam Hospital Providence, RI United States; 9 Division of Infectious Diseases Alpert Medical School of Brown University Providence, RI United States

**Keywords:** criminal justice, incarcerated populations, HIV, acquired immunodeficiency syndrome, mHealth, anti-HIV agents, medication adherence, retention in care, implementation science

## Abstract

**Background:**

Previously incarcerated individuals have suboptimal linkage and engagement in community HIV care. Mobile health (mHealth) interventions have been shown to be effective in addressing these gaps. In Washington, District of Columbia (DC), we conducted a randomized trial of an SMS text messaging–based mHealth intervention (CARE+ Corrections) to increase linkage to community HIV care and antiretroviral treatment adherence among HIV-infected persons involved in the criminal justice system.

**Objective:**

This study aimed to describe the SMS text messaging–based intervention, participant use of the intervention, and barriers and facilitators of implementation.

**Methods:**

From August 2013 to April 2015, HIV-positive incarcerated individuals were recruited within the DC Department of Corrections, and persons released in the past 6 months were recruited within the community via street-based recruitment, community partnerships, and referrals. Participants were followed for 6 months and received weekly or daily SMS text messages. Formative research resulted in the development of the content of the messages in 4 categories: HIV Appointment Reminders, Medication Adherence, Prevention Reminders, and Barriers to Care following release from jail. Participants could customize the timing, frequency, and message content throughout the study period.

**Results:**

Of the 112 participants enrolled, 57 (50.9%) were randomized to the intervention group and 55 (49.1%) to the control group; 2 control participants did not complete the baseline visit, and were dropped from the study, leaving a total of 110 participants who contributed to the analyses. Study retention was similar across both study arms. Median age was 42 years (IQR 30-50), 86% (49/57) were black or African American, 58% (33/57) were male, 25% (14/57) were female, and 18% (10/57) were transgender. Median length of last incarceration was 4 months (IQR 1.7-9.0), and median lifetime number of times incarcerated was 6.5 (IQR 3.5-14.0). Most participants (32/54, 59%) had a baseline viral load of <200 copies/mL. Nearly all participants (52/57, 91%) chose to use a cell phone provided by the study. The most preferred Appointment Reminder message was *Hey how you feeling? Don’t forget to give a call and make your appointment* (19/57, 33%). The most preferred Medication Adherence message was *Don’t forget your skittles!* (31/57, 54%), and 63% (36/57) of participants chose to receive daily (vs weekly) messages from this category at baseline. The most preferred Prevention Reminder message was *Stay strong. Stay clean* (18/57, 32%). The most preferred Barriers to Care message was *Holla at your case manager, they’re here to help* (12/57, 22%). Minor message preference differences were observed among participants enrolled in the jail versus those from the community.

**Conclusions:**

Participants’ ability to customize their SMS text message plan proved helpful. Further large-scale research on mHealth platforms is needed to assess its efficacy among HIV-infected persons with a history of incarceration.

**Trial Registration:**

ClinicalTrials.gov NCT01721226; https://clinicaltrials.gov/ct2/show/NCT01721226

## Introduction

### Background

In 2016, more than 6.6 million adults—or 1 in 38 in the United States—were involved in the criminal justice (CJ) system [1]. The domestic CJ-involved population is disproportionately impacted by HIV, with an estimated HIV prevalence of 1.3% in correctional facilities [2]. In addition, people living with HIV (PLWH) experience high rates of incarceration—an estimated 14% of all persons with HIV are released from a correctional facility annually [3]. Although correctional facilities have been identified as important venues for HIV testing [4-6], HIV treatment [6,7], and reducing HIV-related health disparities [8], PLWH experience poor outcomes along the HIV care continuum after release from correctional facilities. A systematic review illustrated that after release, PLWH had worse linkage to care, retention in care, antiretroviral therapy (ART) receipt, and viral suppression than during incarceration and compared with nonincarcerated populations [9]. Recent evidence demonstrates the utility of technology-based interventions to reach this vulnerable population and to improve ART adherence [10,11].

Ownership of smartphones in the United States has greatly increased from 35% in 2011 to 81% in 2019 [12]. However, smartphone ownership varies by socioeconomic status. For example, smartphone ownership was 71% among persons earning less than US $30,000 per year compared with 95% among persons earning more than US $75,000 per year [12]. Despite these variations, smartphone ownership is increasing and has stimulated the development of mobile health (mHealth) apps to address a wide range of health outcomes from postnatal care [13] to the self-management of long-term illnesses, such as diabetes [14].

A variety of mHealth interventions have been implemented to address HIV/AIDS outcomes among PLWH [15]. Forrest et al [16] proposed a framework that divides mHealth interventions for HIV prevention and care into 3 groups: (1) patients (ie, medication reminders); (2) health systems (ie, evaluation of HIV care delivery and data collection); and (3) populations (ie, mass public health campaigns). However, to date individual-level SMS text messaging remains the primary mode of delivery [15]. A meta-analysis published in 2017 found that interventions with SMS reminders significantly improved HIV appointment attendance, ART adherence, and biological outcomes (ie, CD4 count and HIV viral load) [17]. As a result, there has been substantial interest in developing mHealth interventions to improve HIV outcomes among high-risk populations [18-28].

### Objectives

The National Institute on Drug Abuse funded the Seek, Test, Treat, and Retain (STTR) research initiative to improve the identification, linkage, and engagement in care of HIV-infected vulnerable persons [29]. Within this STTR initiative, 4 studies evaluated mHealth interventions, and a summary of the challenges using mobile phones has been previously reported [10]. In this paper, we characterize the implementation barriers and facilitators for developing an SMS intervention (CARE+ Corrections) among PLWH involved in the CJ system. To assist with future SMS text messaging interventions in this population, we report on lessons learned, share our SMS text message library, discuss optimal timing and frequency of SMS text messaging, and provide training materials to support the introduction of smartphone technology.

## Methods

### Study Design

The CARE+ Corrections study was a randomized, controlled, and longitudinal pilot study in Washington, District of Columbia (DC), and it has been described in detail elsewhere [30-32]. Briefly, the study examined the feasibility and preliminary efficacy of the CARE+ intervention among HIV-infected persons with a history of incarceration. Recruitment occurred in the DC Department of Corrections (DOC) facilities (housing both men and women) and within the community via street-based recruitment, community partnerships, and referrals. Participants recruited in the DOC had anticipated release dates within 6 weeks, whereas participants recruited in the community had been recently released from a jail, prison, or halfway house within the previous 6 months. Study participants were followed for 6 months and outcomes of interest were linkage to HIV care, and achieving HIV viral suppression. To be eligible to receive the intervention, participants needed to pass a basic literacy test to ensure they would be able to read the CARE+ SMS text messages.

The CARE+ intervention was delivered to study participants randomized to the intervention arm and included 2 components: (1) a one-time computerized counseling session called CARE+ Corrections, which was adapted for CJ populations from the CARE+ tool [33,34]; and (2) an SMS text messaging intervention (CARE+ Corrections SMS) that was delivered to study participants in the community. Focusing on the second component in this paper only, CARE+ Corrections SMS comprised daily or weekly scheduled SMS text messages delivered to a cell phone.

Participants were offered the choice of using a basic Android smartphone provided by the study with SMS capability or a monthly US $25 reimbursement to cover texting expenses if they preferred to use their own SMS texting–capable phone. Study phones were provided at no cost to participants (further information about the cell phone plans are detailed elsewhere) [10]. Each participant was allowed 1 replacement phone during the course of the study to account for lost or stolen phones. If a phone was broken and it was deemed not the participant’s fault, that is, a software problem, the participant received a new phone, and this was *not* considered to be part of the one-replacement-phone policy. If participants lost their replacement phones, they were encouraged to find another phone to use for the duration of the study and were provided the monthly US $25 reimbursement.

Study staff set up the SMS intervention on participant’s cell phones using an SMS platform website and completed a registration form that included the participant’s new phone number (or existing one if using their own phone), a participant-chosen nickname (real names were not used to protect privacy of participants), and SMS text message preferences on content and frequency. If participants wanted to make changes to their SMS plan during follow-up visits, a follow-up form was completed by study staff on the SMS platform website.

### SMS Content Development

Formative work conducted in Washington, DC, and Providence, Rhode Island, informed the content of the CARE+ Corrections SMS text messages [30]. The final SMS library included 4 categories with 9 prewritten messages and the ability to customize a message within each category. (See Multimedia Appendix 1 for the full SMS text message library.) We addressed the following 4 content categories.

#### HIV Appointment Reminders

Messages focused on reminding participants to attend their prescheduled HIV care appointment or reminded participants to schedule a new appointment.

#### Medication Adherence

This comprised message reminders to take their HIV medications. Messages varied from highlighting the importance of medications to, *keep your body strong and healthy* to reaching out to caseworkers to ask for help with medication adherence.

#### HIV Prevention Reminders

Participants chose to receive a message on safe sex practices or tips and mantras to avoid substance use.

#### Barriers to Care

Messages focused on areas participants may need help with when leaving the correctional system, such as finding housing, employment, etc. This category was adapted from the formative work to include specific resources found in Washington, DC, eg, providing the actual phone number of the office that helps returning citizens find employment within the message.

CARE+ Corrections study staff worked with an SMS vendor (Dimagi, Cambridge, MA) to create an SMS platform for automated text messaging. The initial SMS platform delivered messages from all 4 content areas in a single message thread at a preset frequency and in a single communication. Before study initiation, study staff members conducted pilot testing of the platform and concluded that greater flexibility in the frequency of messaging was required. Staff members indicated that receiving all messages in a single thread led to message fatigue and content was often overlooked, given multiple messages needed to be read at the same time. In response to this feedback, the SMS vendor was able to adapt the platform to allow participants to choose the frequency of messaging (daily versus weekly), timing of messaging (eg, am or pm), and ability to change messaging content in each category (Table 1), with slight variations in 2 message content categories: HIV Appointment Reminder and Barriers to Care.

Frequency for the HIV appointment reminder message depended on the date of the participant’s appointment and the Barriers to Care message was only available once per week to participants during the first month of the intervention or, if reincarcerated during the study period, 1 month following reentry into the community.

**Table 1:** SMS message content details, customization, time options, and frequency.

Message category	Able to write own custom text message	Time options	Able to customize text message frequency options	Able to change options during follow-up
HIV appointment reminder	✓^a^	8:00 am 10:00 am 12:00 pm 2:00 pm 5:00 pm 8:00 pm	X^b^ Message sent on the basis of appointment date: 19, 14, 7, 3, 2, and 1 day before HIV appointment date	✓
Medication adherence	✓	8:00 am 10:00 am 12:00 pm 2:00 pm 5:00 pm 8:00 pm	✓ Choice of daily or weekly option	✓
Prevention reminder	✓	8:00 am 10:00 am 12:00 pm 2:00 pm 5:00 pm 8:00 pm	✓ Choice of daily or weekly option	✓
Barriers to care	✓	X	X Message sent weekly during the first month of the intervention or if reincarcerated, one month following reentry into the community	X

^a^✓: Yes, participants were able to customize this area

^b^X: No, participants were unable to customize this area.

### Data Analysis

We generated descriptive statistics (eg, frequency, mean, and/or median) of characteristics of study participants in the intervention arm and their message preferences (content, time, and frequency) throughout the study using SAS version 9.4 (SAS Institute Inc, Cary, NC, USA).

### Human Subjects Review

The George Washington University and The Miriam Hospital Institutional Review Boards approved the CARE+ Corrections Study and the US Office of Human Research Protections reviewed it.

## Results

### Demographics

Of 219 persons assessed for eligibility, 112 (51.1%) were enrolled and randomized. Of those enrolled, 57 (50.9%) were randomized to the CARE + Corrections intervention group and 55 (49.1%) to the control group; two control participants did not complete the baseline visit, and were therefore dropped from the study, leaving a total of 110 participants who contributed to the analyses. Study retention was similar across both study arms. Although 41 of 110 (37.3%) experienced reincarceration during the 6-month follow-up period, 96 of 110 (87.3%) completed all three study visits. This paper will focus on the experience of the 57 individuals randomized to the intervention group (Table 2).

Most participants (37/57, 65%) were enrolled in the community after recent release from a correctional facility. The median age was 42 years (IQR 30-50). Most (49/57, 86%) were black or African American and male (33/57, 58%), with 25% (14/57) being female and 18% (10/57) being male-to-female transgender. The participants’ median length of last incarceration was 4 months (IQR 1.7-9.0), and the median number of times of being incarcerated throughout their lifetime was 6.5 times (IQR 3.5-14.0).

**Table 2:** Characteristics of intervention arm (n=57) at baseline.

Characteristic		Values
**Enrollment location,** **n (%)**		
	Community	37 (65)
	District of Columbia Department of Corrections	20 (35)
**Gender,** **n (%)**		
	Male	33 (58)
	Female	14 (25)
	Transgender (male to female)	10 (18)
**Race/ethnicity,** **n (%)**		
	Non-Hispanic black/African American	49 (86)
	Non-Hispanic white	2 (3)
	Other	6 (11)
Age (years), median (IQR)		42 (30-50)
**Sexual orientation,** **n (%)**		
	Heterosexual or straight	45 (78)
	Homosexual, gay, or lesbian	6 (11)
	Bisexual	6 (11)
**Education,** **n (%)**		
	≤High school	49 (86)
	>High school	8 (14)
**Housing stability,** **n (%)**		
	Stable	45 (79)
	Unstable	12 (21)
**Drug dependence (Texas Christian University score)^a^, n (%)**		
	No (9-11)	25 (44)
	Yes (12-18)	32 (56)
**Ever injection drug use** **,** **n (%)**		
	No	47 (82)
	Yes	10 (18)
**Injection drug use in the 3 months before last incarceration^b^, n (%)**		
	No	6 (60)
	Yes	4 (40)
**Ever noninjection drug use,** **n (%)**		
	No	9 (16)
	Yes	48 (84)
**Noninjection drug use in 3 months before last incarceration^c^, n (%)**		
	No	15 (31)
	Yes	33 (69)
**Depressive symptoms (Center for Epidemiological Studies Depression Scale-10)** **,** **n (%)**		
	No	29 (51)
	Yes	28 (49)
Length of last incarceration (months), median (IQR)		4 (1.7-9.0)
Number of times in jail/prison, lifetime^c^, median (IQR)		6.5 (3.5-14.0)
**Study baseline viral load^d^, n (%)**		
	<200 copies/mL	32 (59)
	≥200 copies/mL	22 (41)
**Cell phone choice** **,** **n (%)**		
	CARE+ phone	52 (91)
	Personal phone	5 (9)

^a^12 months before last incarceration.

^b^Among 10 participants reporting ever injection drug use.

^c^Among 48 participants reporting ever noninjection drug use.

^d^Among 54 participants.

### Implementation Logistics at Study Start-Up

#### Cell Phone Logistics

Most participants (52/57, 91%) chose to use a cell phone provided by the study (Table 2); of those participants, nearly two-thirds (35/52, 67%) required a replacement phone during the follow-up period. Most replacement phones (30/35, 86%) were provided because of reported loss or theft of the phone. Only 14% (5/35) of replacement phones were provided because of a faulty phone and did not count toward the participant’s one-replacement-phone policy. At the end of the study, participants were asked to return their study phones; however, more than 90% (47/52) of participants kept the phone provided by the study (they reported not having their phone at the final visit).

#### Service Interruptions and Billing/Overage Issues

As nearly two-third of the phones provided by the study had to be replaced because of loss or theft, many were left without service while waiting for a replacement phone. In addition, reincarceration led to service disruptions. To avoid intervention interruptions because of phone issues, study staff had regular clinic hours in a known location throughout the duration of the study and participants knew to drop-in regarding any issues with phones. Furthermore, participants knew which community partners were affiliated with the CARE+ study, and study staff would receive calls from participants at these community partner locations to schedule appointments for phone replacements.

We used a pooled minutes cell phone plan, in which all phone lines shared available minutes, to account for some participants using more minutes and others less. Using this model, the study never went over its total allotted monthly minutes. Several participants exceeded their monthly minute allotments, and if the amount was significant, study staff would call the participant and review the participant’s cell phone plan. In 2 instances, participants used smartphone services that incurred additional fees (eg, downloading apps and calling 411 for information). In both cases, study staff worked with participants to call one of the community partners instead for information and inform participants that downloading apps on the study phone was not allowed. In addition, study staff worked with the phone carrier to limit the downloading of apps on study phones.

#### Mobile Health in an Older Population

As the median age of CARE+ Corrections participants was 42 years, study staff recognized that cell phone training for smartphones would be required for some study participants. During the initial session, study staff assessed participants’ knowledge and ability in using the smartphones provided by the study and found that the majority of participants required 1-on-1 training before the initiation of the intervention. Study staff developed a 5- to 10-min cell phone training module that was delivered during the baseline visit (see Multimedia Appendix 2). The training module was interactive and included instructions on how to turn the phone on and off, entering information for contacts, opportunities to practice sending SMS text messages and making telephone calls, and instructions for using the talk-to-text option. We provided ongoing support at all follow-up appointments, including reviewing the training again at in-person visits.

### Participant Preferences: Message Content and Preferred Frequency

Participants had the option of customizing the content, timing, and frequency of the SMS text messages by the 4 topic areas.

#### HIV Appointment Reminder Message Preferences

The most popular message chosen at baseline for the HIV appointment reminder message (see Table 3) was, *Hey how you feeling? Don’t forget to give a call and make your appointment* (19/57, 33%), followed by *Don’t forget your appointment – it’s important* (11/57, 19%) and *You’re worth it – remember your clinic appointment* (8/57, 14%).

A total of 3 participants created custom message content at baseline, such as, *Dr. [name] on [date]* (see Multimedia Appendix 3 for other custom messaging). The most popular message time chosen at baseline was 8:00 am (20/57, 35%), followed by 12:00 pm (12/57, 21%). During follow-up, 28% (16/57) and 14% (8/57) of participants made changes to message content and time, respectively. The most popular message content changes during follow-up were to choose, *Don’t forget your appointment – it’s important* (5/17, 29%) and *Your health comes first – go to your appointment* (5/17, 29%). The most popular message time changes during follow-up were to choose 8:00 am (3/9, 33%) and 10:00 am (3/9, 33%).

**Table 3:** HIV appointment reminder messaging.

Messaging options, HIV appointment reminder		Values, n (%)
**Message content**		
	*Hey how you feeling? Don’t forget to give a call and make your appointment* ^a^	*19 (33)*
	*Don’t forget your appointment – it’s important*	*11 (19)*
	*You’re worth it – remember your clinic appointment*	*8 (14)*
	Your doctor wants you to come to your appointment	5 (9)
	Your health comes first – go to your appointment	4 (7)
	Your doctors are here to help you –go to your appointment	4 (7)
	Custom message content	3 (5)
	Call your case manager – he/she can help you get to clinic	2 (4)
	Going to the clinic helps you stay healthy	1 (2)
	Can’t remember when your next appointment is? Call the clinic to find out	0 (0)
	Total	57 (100)
**Message time**		
	*8:00 am*	*20 (35)*
	*12:00 pm*	*12 (21)*
	10:00 am	11 (19)
	5:00 pm	6 (11)
	2:00 pm	4 (7)
	8:00 pm	4 (7)
	Total	57 (100)

^a^Italics indicate the 3 most popular messages and 2 most popular message time options at baseline.

#### Medication Adherence Message Preferences

The most popular message chosen at baseline was *Don’t forget your skittles!* (31/57, 54%), followed by, *Meds keep your body strong and healthy* (10/57, 18%) and *Hey, take your vitamins!* (7/57, 12%; Table 4).

A participant created a custom message at baseline and wanted us to include a smiley face within the message, *Hey [name] don't forget those mones! :)* The most popular message times chosen at baseline were 10:00 am (17/57, 30%) and 8:00 am (15/57, 26%). At baseline, most participants chose daily message frequency (36/57, 63%). During follow-up, 21% (12/57), 18% (10/57), and 14% (8/57) of participants made changes to message content, time, and frequency, respectively. The most popular message content change during follow-up was to create custom message content (3/12, 25%). The most popular message frequency change during follow-up was from weekly to daily (7/8, 88%).

**Table 4:** Medication adherence messaging.

Messaging options, medication adherence		Values, n (%)
**Message content**		
	*Don’t forget your skittles!* ^a^	*31 (54)*
	*Meds keep your body strong and healthy*	*10 (18)*
	*Hey, take your vitamins!*	*7 (12)*
	You got to play to win. So don’t forget your meds	3 (5)
	The best way to stay healthy is to take your meds on time and the right way	2 (4)
	Give meaning to your life … Now!	2 (4)
	Custom message content	1 (2)
	Your meds may not work anymore if you forget to take them	1 (2)
	Adherence to meds means taking the right dose at the right time	0 (0)
	Call your case manager—he/she can help you find ways to remember to take your meds	0 (0)
	Total	57 (100)
**Message time**		
	*10:00 am*	*17 (30)*
	*8:00 am*	*15 (26)*
	8:00 pm	14 (25)
	5:00 pm	4 (7)
	2:00 pm	4 (7)
	12:00 pm	3 (5)
	Total	57 (100)
**Message frequency**		
	*Daily*	*36 (63)*
	Weekly	21 (37)
	Total	57 (100)

^a^Italics indicate the 3 most popular messages, 2 most popular message time options, and most popular message frequency option at baseline.

#### Prevention Reminder Message Preferences

The most popular message chosen at baseline was, *Stay strong. Stay clean* (18/57, 32%), followed by *Safe sex is important. Use a condom* (8/57, 14%) and *Be smart. Use a condom* (7/57, 12%; Table 5).

A total of 2 participants created custom content at baseline (see Multimedia Appendix 3). For 1 participant, the custom message of *Keep your eyes on your own work* was a saying his father used often to help the participant remember to stay focused on what matters.

When the messages were categorized as substance use prevention, safe sex, or custom content, most participants (31/57, 54%) chose substance use content. The most popular message times chosen at baseline were 8:00 am (13/57, 23%) and 8:00 pm (12/57, 21%). At baseline, most participants chose weekly message frequency (31/57, 54%). During follow-up, 26% (15/57), 28% (16/57), and 18% (10/57) of participants made changes to message content, time, and frequency, respectively. The most popular message time change during follow-up was to 10:00 am (7/18, 39%). The most popular message frequency change during follow-up was from weekly to daily (9/13, 69%).

**Table 5:** Prevention reminder messaging.

Messaging options, prevention reminders		Values, n (%)
**Message content**		
	*Stay strong. Stay clean* ^a,b^	*18 (32)*
	*Safe sex is important. Use a condom* ^c^	*8 (14)*
	*Be smart. Use a condom* ^c^	*7 (12)*
	One day at a time. Just for today, don’t use^b^	6 (10)
	Did you read “Get your Freak on for Dummies”—it says you must wear a rubber!^c^	5 (9)
	Staying clean is most important. Call your case manager for help^b^	4 (7)
	If you are using, you may forget your meds^b^	3 (5)
	Don’t forget to wrap it or don’t give it up!^c^	3 (5)
	Custom message content	2 (4)
	Protect yourself and your partner. Use a condom^c^	1 (2)
	Total	57 (100)
**Message time**		
	*8:00 am*	*13 (23)*
	*8:00 pm*	*12 (21)*
	2:00 pm	11 (19)
	10:00 am	10 (18)
	5:00 pm	7 (12)
	12:00 pm	4 (7)
	Total	57 (100)
**Message frequency**		
	*Weekly*	*31 (54)*
	Daily	26 (46)
	Total	57 (100)

^a^Italics indicate the 3 most popular messages, 2 most popular message time options, and most popular message frequency option at baseline.

^b^Substance use prevention content.

^c^Safe sex content.

#### Barriers to Care Message Preferences

The most popular messages chosen at baseline for the Barriers to Care messaging (Table 6) were *Holla at your case manager, they’re here to help* (12/57, 21%), *Hey! Stay linked to your clinic so you can get your meds and care* (9/57, 16%), *Get help for your housing: call (XXX) XXX-XXXX [Local CBO that helps with housing]* (9/57, 16%), and *Check on job and training programs today* (9/57, 16%).

More participants chose to create custom content for this message category compared with the other categories (see Multimedia Appendix 3). For example, a custom message, *Hey don’t forget your parole appointment on [date],* highlighted the importance of incarceration-related priorities and other custom messages focused on positivity, such as *Keep hope alive!, We love you!* and *Stay positive!* The most popular message times chosen at baseline were 10:00 am (17/57, 30%) and 8:00 am (16/57, 28%).

**Table 6:** Barriers to care messaging.

Messaging options, barriers to care		Values, n (%)
**Message content**		
	*Holla at your case manager, they’re here to help* ^a,b,c^	*12 (21)*
	*Hey! Stay linked to your clinic so you can get your meds and care* ^b^	*9 (16)*
	*Get help for your housing: call xxx-xxx-xxxx* ^b,c^	*9 (16)*
	*Check on job and training programs today* ^c^	*9 (16)*
	Remember to get a case manager: call xxx-xxx-xxxx	7 (12)
	Custom message content	5 (9)
	Get help getting your entitlement/insurance programs: call xxx-xxx-xxxx	3 (5)
	Can’t get your prescriptions? Call your clinic or case manager	2 (3)
	Need a ride to your appointment? Call your case manager at xxx-xxx-xxxx	1 (2)
	Call transportation services so you can get to your clinic visits: call xxx-xxx-xxxx	0 (0)
	Total	57 (100)
**Message time**		
	*10:00 AM*	*17 (30)*
	*8:00 AM*	*16 (28)*
	2:00 PM	10 (18)
	8:00 PM	7 (12)
	5:00 PM	4 (7)
	12:00 PM	3 (5)
	Total	57 (100)

^a^Italics indicate the 3 most popular messages and 2 most popular message time options at baseline.

^b^The 3 most popular message content options among those enrolled in the community.

^c^The 3 most popular message content options among those enrolled in the District of Columbia Department of Corrections.

### Results by Enrollment Site

There were minor differences in message preferences between those enrolled in the community (released from a correctional facility within the last 6 months) versus those enrolled in the DC DOC. For the prevention reminder messaging, the most popular messages chosen at baseline among those enrolled in the community were *Stay strong. Stay clean* (9/37, 24%), *Safe sex is important. Use a condom* (5/37, 14%), *Be smart. Use a condom* (5/37, 14%), and *One day at a time. Just for today, don’t use* (5/37, 14%). The most popular messages chosen at baseline among those enrolled in jail were *Stay strong. Stay clean* (9/20, 45%), *Safe sex is important. Use a condom* (3/20, 15%), and *Did you read “Get your Freak on for Dummies*”—*it says you must wear a rubber!* (3/20, 15%). Among the barriers to care messages, the most popular messages chosen at baseline among those enrolled in the community were, *Holla at your case manager, they’re here to help* (8/37, 22%), *Hey! Stay linked to your clinic so you can get your meds and care* (7/37, 19%), and *Get help for your housing: call (XXX) XXX-XXXX* (5/37, 14%). Participants enrolled in the jail were more likely to choose *Check on job and training programs today* (5/20, 25%), *Holla at your case manager, they’re here to help* (4/20, 20%), and *Get help for your housing: call (XXX) XXX-XXXX* (4/20, 20%).

## Discussion

### Principal Findings

HIV-infected individuals with a history of incarceration represent a vulnerable community in need of innovative interventions to address many barriers to HIV care and adherence to ART. We were able to implement an SMS-based intervention and report lessons learned for implementation and message preferences. This knowledge will be invaluable to others delivering SMS interventions in this vulnerable population.

### Lessons Learned

As reported elsewhere [10], challenges implementing mHealth technologies for CJ-involved population include service interruptions, billing/overage issues, and users’ experience with an SMS text messaging platform that is automated. Adding to this knowledge, during implementation of CARE+ SMS, we learned the following: (1) the importance of pilot testing the intervention and adapting the intervention for the population; (2) cell phone implementation considerations; and (3) providing an array of message delivery preferences (ie, frequency and timing).

#### Pilot Testing and Adapting to Your Population

The study team saw huge improvements to the CARE+ Corrections intervention after pilot testing the intervention. Recognizing the significance of customization (ie, timing and frequency) of messaging to avoid message fatigue, the study team was able to make changes to the SMS platform before intervention implementation to provide study participants more flexibility.

Adapting the SMS text message library to accurately reflect the common experiences for the population was essential. For example, study participants were more likely to choose a substance use message versus the safe sex message under the Prevention Reminder category. This reflected what has been previously observed among incarcerated persons facing a 3- to 8-fold increased risk of drug-related death, 1 to 2 weeks following release compared with 3 to 12 weeks following release [35]. Future interventions should review the literature, engage with community-based organizations (CBOs), and conduct formative research with the population to inform message development to identify unique barriers and resources for the population of interest.

In addition, as our study population was older, adapting the implementation strategy to meet the needs of older participants was essential to effective implementation of the SMS text message plan. On the basis of previous literature [10,36,37], we recognized that additional cell phone training would be required for some study participants and developed a simple in-person training (see Multimedia Appendix 2). This was consistent with previous literature, in which a 2016 review of designing, implementing, and evaluating mHealth solutions among older adults highlighted the importance of ease of use of the mobile platform and an understanding of technical literacy of the user [38]. Furthermore, in-person contact helped to facilitate familiarizing the older population with mobile platforms. In our study, participants received 1-on-1 support with the initial setup of the CARE+ SMS program during their baseline visit. Another option for future mHealth interventions among older populations would be to use YouTube videos to further build rapport among study participants and build mobile skills [39].

#### Cell Phone Implementation Considerations

The Android smartphones provided by the study were highly desirable; however, participants’ chaotic personal environments impacted smartphone retention. Most CARE+ participants opted to receive the phone provided by the study; however, most required a replacement phone and almost all kept their phone at the end of the study. In contrast, another STTR study reported 100% of users discarded the inexpensive, older model flip phone [10]. Future mHealth interventions, if possible, should continue to provide smartphones but consider budgeting more funds for cell phone replacement, given the likelihood of phone replacements. If the budget to provide a cell phone is not available and/or sustainable, an idea for future interventions would be to use Web-based telephone services (ie, Google Voice and WhatsApp) to continue sending SMS text messages via email when cell phone service is turned off, as was used in another STTR site [10].

Leveraging community partnerships proved very useful to avoid cell phone service interruptions because of lost or faulty phones. In addition to having regular office/study hours at the CARE+ community site, CARE+ staff identified contacts at multiple popular CBOs to provide participants with options for study engagement. Participants knew which community partners were affiliated with CARE+ and study staff would receive calls from participants at these community partner locations to set up appointments for phone replacements. This also proved useful for study retention purposes*.*

Identifying economic solutions with the cell phone carrier reduced economic burden on study budget. Using the pooled minutes approach provided the much-needed flexibility for CARE+ study participants, as some used very little of the suggested minutes allotted per month whereas others went over consistently. Future interventions should consider the pooled minutes approach to avoid overage issues and work with carriers to turn off specific apps and phone options that could incur monthly charges. Furthermore, incorporating cost-effectiveness analyses using templates [40], such as the one used by Reback et al [41] to evaluate a substance use and HIV risk reduction SMS intervention, will provide useful information for the feasibility of mHealth interventions at the population level.

#### Message Frequency and Customization

Previous research has indicated that SMS interventions with 1 or more daily messages demonstrated *smaller* effects than interventions that only sent messages weekly [42]; however, CARE+ participant preferred more frequent SMS text messages. At baseline, participants were more likely to choose to receive the message every day instead of once per week. Furthermore, during follow-up, a large proportion of participants changed their message frequency option from weekly to daily.

Few chose to create their own message. We believe this could reflect the success and importance of the formative work, adequately reflecting the messages they wanted to receive. However, even with few selecting to create their own message, providing this as an option is important, as interventions that allow for message customization are more effective at promoting adherence to ART than those that send uniform messages to all participants [42].

For those who did create custom messages (see Multimedia Appendix 3), they provided us insights into missed messaging opportunities for future SMS interventions for persons with a history of incarceration. For example, a custom message reminded the participant of the participant’s parole appointment and thus highlighted the potential of adding community supervision—related reminder options to the Barriers to Care content area. Highlighting CJ status within the message was in contrast to our initial study goals of avoiding mentioning either HIV or CJ status. Future research is needed to explore the breadth and effectiveness of CJ-focused messaging.

#### Differences by Enrollment Site

Participants who were enrolled in the jail chose different Barriers to Care messaging compared with those participants enrolled in the community—highlighting the differences in barriers encountered at 2 different time points (immediate release versus up to 6 months before release from jail). The popularity of messages about seeking housing and job training programs among jail enrollees versus community-enrolled participants could be because of the fact that the latter may have had more time in the community to address these needs. This is supported by the literature, with persons immediately released from correctional facilities reporting transitional challenges, such as not knowing how to find shelter and feeling dumped into the city, unsure where to spend their first night [43]. Immediate needs following reentry can also vary by gender, with men reporting finding a job and education as most important immediately, whereas women identified shelter and substance abuse as their top priorities [44]. Future interventions should consider the timing of release from a correctional setting and choose Barriers to Care messaging that reflects the timing of release.

### Limitations

Although this study provides important details for future mHealth interventions among this vulnerable population, this study had several limitations. First, this study reported on the intervention arm of a pilot feasibility study. Given the small sample size, we lacked that statistical power to make between-group comparisons (eg, gender, race, and enrollment site), limiting our ability to inform mHealth interventions among specific populations. In addition, our SMS platform website (Dimagi) was a 1-way text service; thus, we could not evaluate engagement in the SMS intervention or confirm receipt of the SMS text messages. Furthermore, this study was limited to programming data (from Dimagi) and the experiences of study staff. Future mHealth intervention studies would benefit from larger sample sizes to evaluate messaging preference among various sociodemographic variables and qualitative research to better understand the specifics of SMS interventions that work well for the target population.

### Conclusions

In this paper, we report the implementation of an SMS intervention for HIV-infected persons with a history of incarceration. Highlighting the implementation of a real-world application of an mHealth platform, subsequent programs working with the same or other vulnerable populations can use the findings, methodology, and trainings to implement and benefit from our lessons learned.

## Appendix

Multimedia Appendix 1 CARE+ Corrections SMS Messaging Library.

Multimedia Appendix 2 How to Use Your Droid 4.

Multimedia Appendix 3 CARE+ Customized Messages at Baseline.

Multimedia Appendix 4 CONSORT-EHEALTH checklist (V 1.6.1).

## Abbreviations

ART: antiretroviral therapy
CBO: community-based organization
CJ: criminal justice
DC: District of Columbia
DOC: Department of Corrections
mHealth: mobile health
PLWH: people living with HIV
STTR: Seek, Test, Treat, and Retain
UNC: University of North Carolina
