# Supplementary material for: The Implementation of a Text Messaging Intervention to Improve HIV Continuum of Care Outcomes Among Persons Recently Released From Correctional Facilities: Randomized Controlled Trial
Source: JMIR Mhealth Uhealth. 2020 Feb 13;8(2):e16220. doi: 10.2196/16220 (PMC7055782; doi:10.2196/16220)
Supplement: Multimedia Appendix 1 [file mhealth_v8i2e16220_app1.docx]

**Appendix 1: CARE+ Corrections SMS Messaging Library**

| **Category** | **Message options** |
| --- | --- |
| HIV Appointment Reminder Messaging | - Hey how you feeling? Don’t forget to give a call and make your appointment - You’re worth it – remember your clinic appointment - Your doctors are here to help you –go to your appointment - Call your case manager – he/she can help you get to clinic - Don’t forget your appointment – it’s important - Your health comes first – go to your appointment - Can’t remember when your next appointment is? Call the clinic to find out. - Your doctor wants you to come to your appointment - Going to the clinic helps you stay healthy - Participant-created message |
| Medication Adherence Messaging | - Meds keep your body strong and healthy. - Don’t forget your skittles! - The best way to stay healthy is to take your meds on time and the right way - Adherence to meds means taking the right dose at the right time - Your meds may not work anymore if you forget to take them - You got to play to win. So don’t forget your meds. - Call your case manager—he/she can help you find ways to remember to take your meds - Give meaning to your life ... Now! - Hey, take your vitamins! - Participant-created message |
| Prevention Reminder Messaging | - Safe sex is important. Use a condom. - Don’t forget to wrap it or don’t give it up! - Did you read “Get your Freak on for Dummies”—it says you must wear a rubber! - Be smart. Use a condom. - Protect yourself and your partner. Use a condom. - If you are using, you may forget your meds. - One day at a time. Just for today, don’t use. - Stay strong. Stay clean. - Staying clean is most important. Call your case manager for help. - Participant-created message |
| Barriers to Care Messaging (sent to participant between registration and first check-in appointment; and sent again if person is re-incarcerated) | - Remember to get a case manager: call xxx-xxx-xxxx. - Holla at your case manager, they’re here to help - Hey! Stay linked to your clinic so you can get your meds and care. - Need a ride to your appointment? Call your case manager at xxx-xxx-xxxx - Can’t get your prescriptions? Call your clinic or case manager. - Get help for your housing: call xxx-xxx-xxxx - Call transportation services so you can get to your clinic visits: call xxx-xxx-xxxx - Check on job and training programs today - Get help getting your entitlement/insurance programs: call xxx-xxx-xxxx - Participant-created message |
| Welcome message sent during the week after registration | - Welcome to the CARE study! We appreciate your participation. Call us at 800-xxx-CARE (2273) if you have any questions. |
| Monthly message reminding participants to schedule their monthly check-in with study staff | - We want to talk to you! Please call 800-xxx-CARE (2273) to be sure your monthly check-in is scheduled. |
