# Supplementary figures and images for: The Implementation of a Text Messaging Intervention to Improve HIV Continuum of Care Outcomes Among Persons Recently Released From Correctional Facilities: Randomized Controlled Trial
Source: JMIR Mhealth Uhealth. 2020 Feb 13;8(2):e16220. doi: 10.2196/16220 (PMC7055782; doi:10.2196/16220)

**Appendix 2: How to Use Your Droid 4**


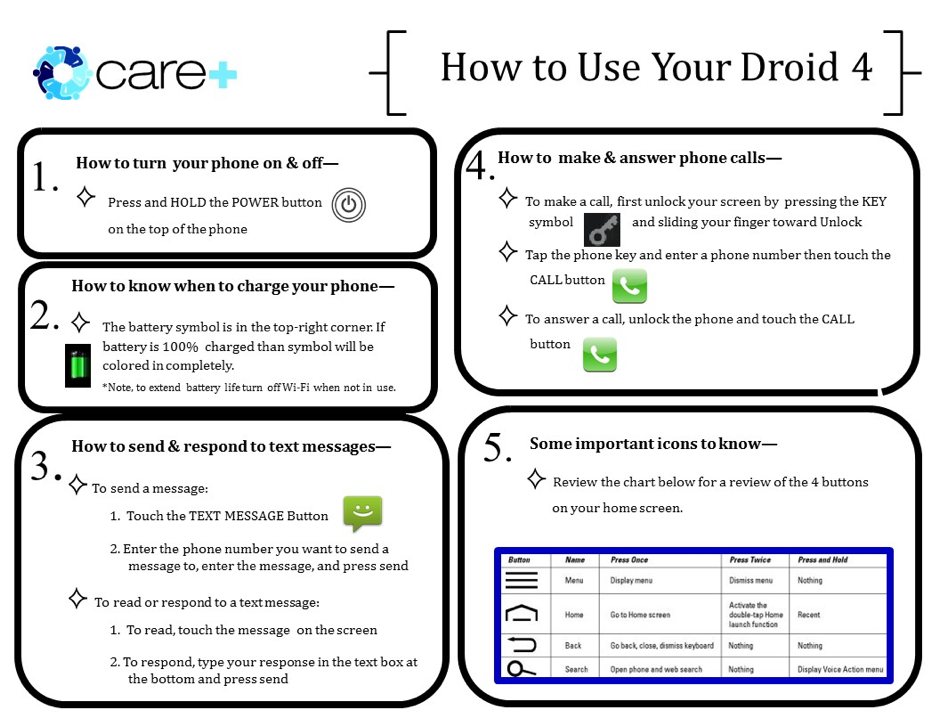

Supplement: Multimedia Appendix 2 [file mhealth_v8i2e16220_app2.docx]
