# Supplementary material for: The Implementation of a Text Messaging Intervention to Improve HIV Continuum of Care Outcomes Among Persons Recently Released From Correctional Facilities: Randomized Controlled Trial
Source: JMIR Mhealth Uhealth. 2020 Feb 13;8(2):e16220. doi: 10.2196/16220 (PMC7055782; doi:10.2196/16220)
Supplement: Multimedia Appendix 3 [file mhealth_v8i2e16220_app3.docx]

**Appendix 3: CARE+ Customized Messages at Baseline**

- **Appointment reminder**
  - “Dr. [name] on [date]”
  - “Remember to call us when you schedule your next appointment”
  - “You have an appointment with Dr. [name] on [date]”
- **Medication adherence**
  - “Hey [name] don't forget those mones! :)”
- **Prevention reminder**
  - “Keep your eyes on your own work.”
  - “Stay drug free.
- **Barriers to care**
  - “Don’t forget kids on Monday”
  - “Hey don't forget your parole appointment on [date].”
  - “Keep hope alive!”
  - “Stay positive!”
  - “We love you.”

**Custom Messages Created During Follow-up**

- **Medication adherence**
  - “Hey [initial]! It's your med time! Don't forget to take it!”
  - “You know what to do!!!!!!!!!!!!!!!!!!!!!!”
  - “Remember to make an appointment for the doctor! :)”
- **Prevention reminder**
  - “Hey [name] don't flip up!”
  - “Make sure to use protection”
  - “Protect yourself.”
